# Supplementary material for: Genetic and pharmacologic alterations of claudin9 levels suffice to induce functional and mature inner hair cells
Source: bioRxiv. 2023 Oct 10:2023.10.08.561387. Preprint. [Version 1] doi: 10.1101/2023.10.08.561387 (PMC10592694; doi:10.1101/2023.10.08.561387)

bioRxiv preprint doi: https://doi.org/10.1101/2023.10.08.561387; this version posted October 10, 2023. The copyright holder for this preprint (which was not certified by peer review) is the author/funder, who has granted bioRxiv a license to display the preprint in perpetuity. It is made available under aCC-BY 4.0 International license.

Supplement S1

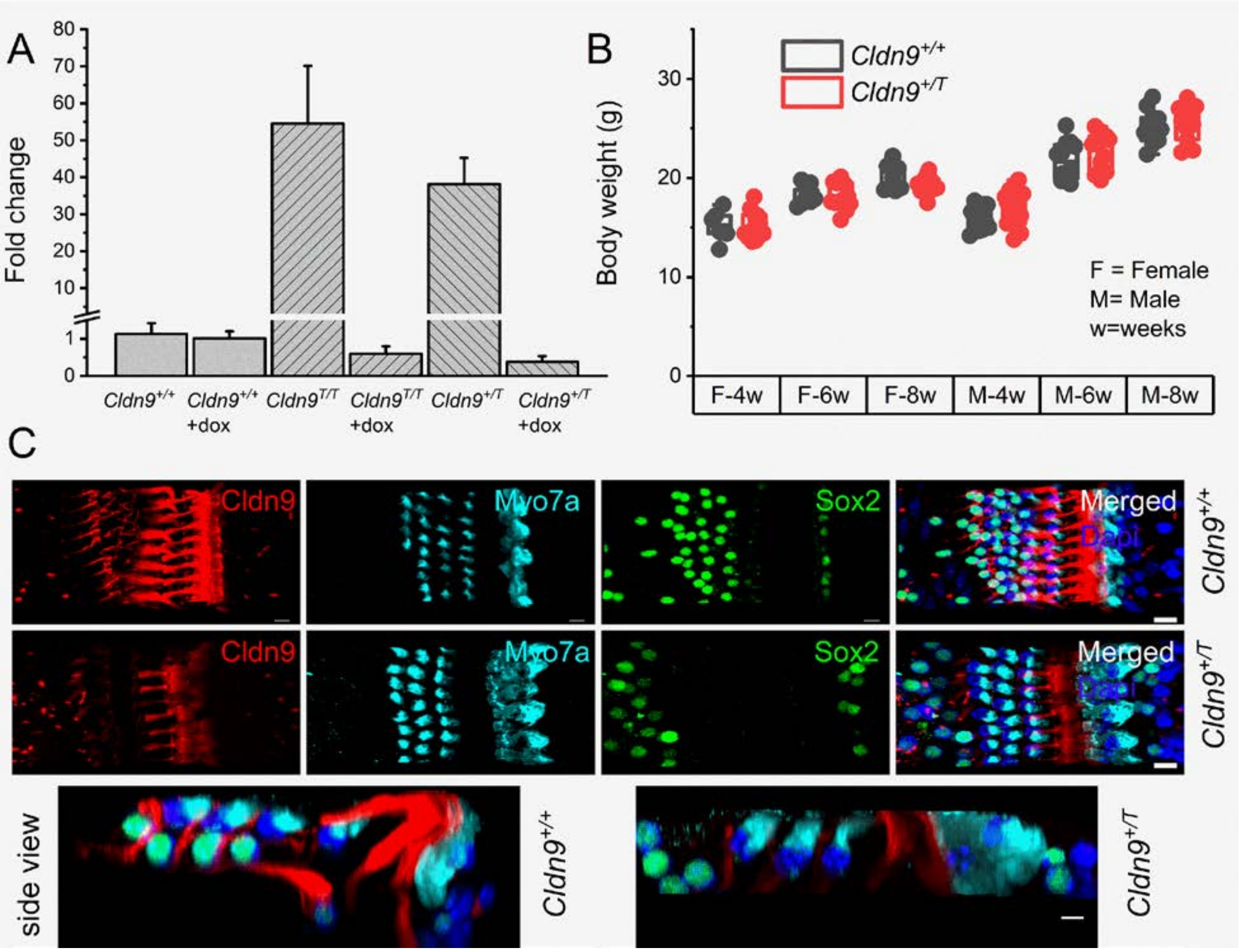

bioRxiv preprint doi: https://doi.org/10.1101/2023.10.08.561387; this version posted October 10, 2023. The copyright holder for this preprint (which was not certified by peer review) is the author/funder, who has granted bioRxiv a license to display the preprint in perpetuity. It is made available under aCC-BY 4.0 International license.

Supplement S2

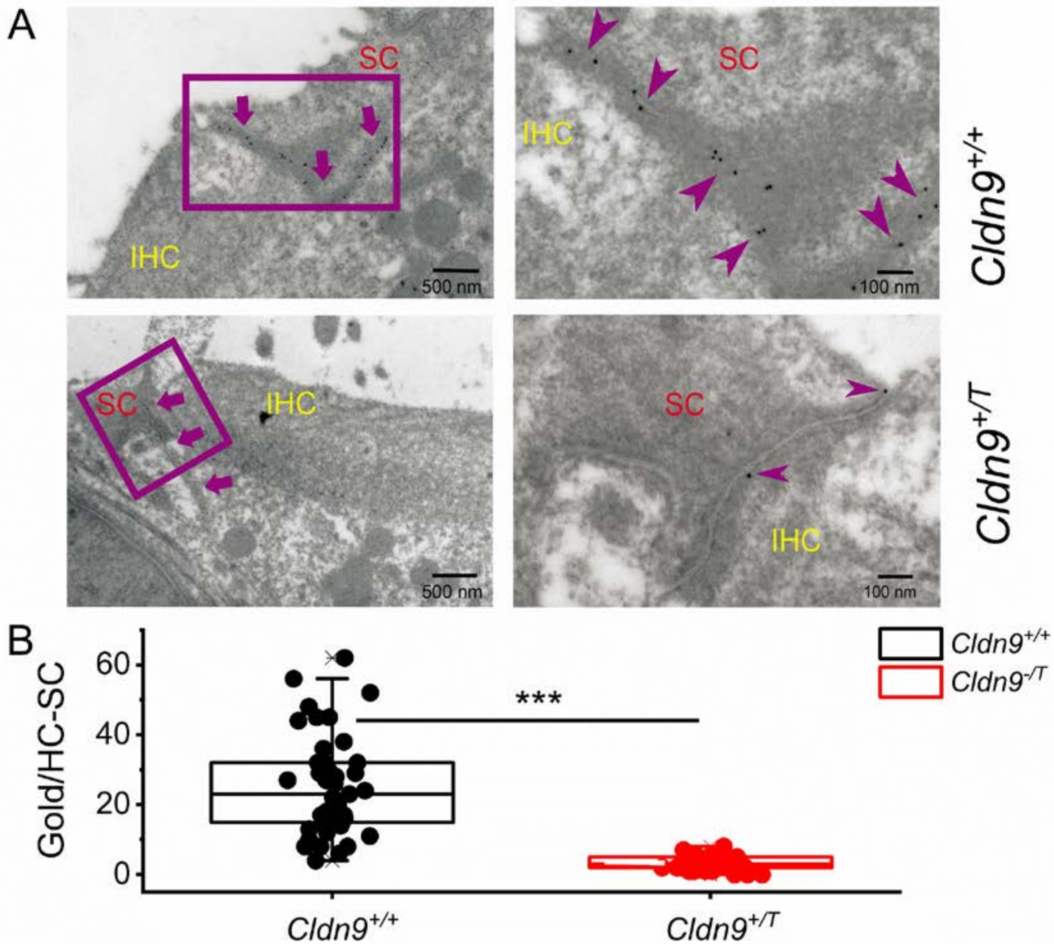

bioRxiv preprint doi: https://doi.org/10.1101/2023.10.08.561387; this version posted October 10, 2023. The copyright holder for this preprint (which was not certified by peer review) is the author/funder, who has granted bioRxiv a license to display the preprint in perpetuity. It is made available under aCC-BY 4.0 International license.

Supplement S3

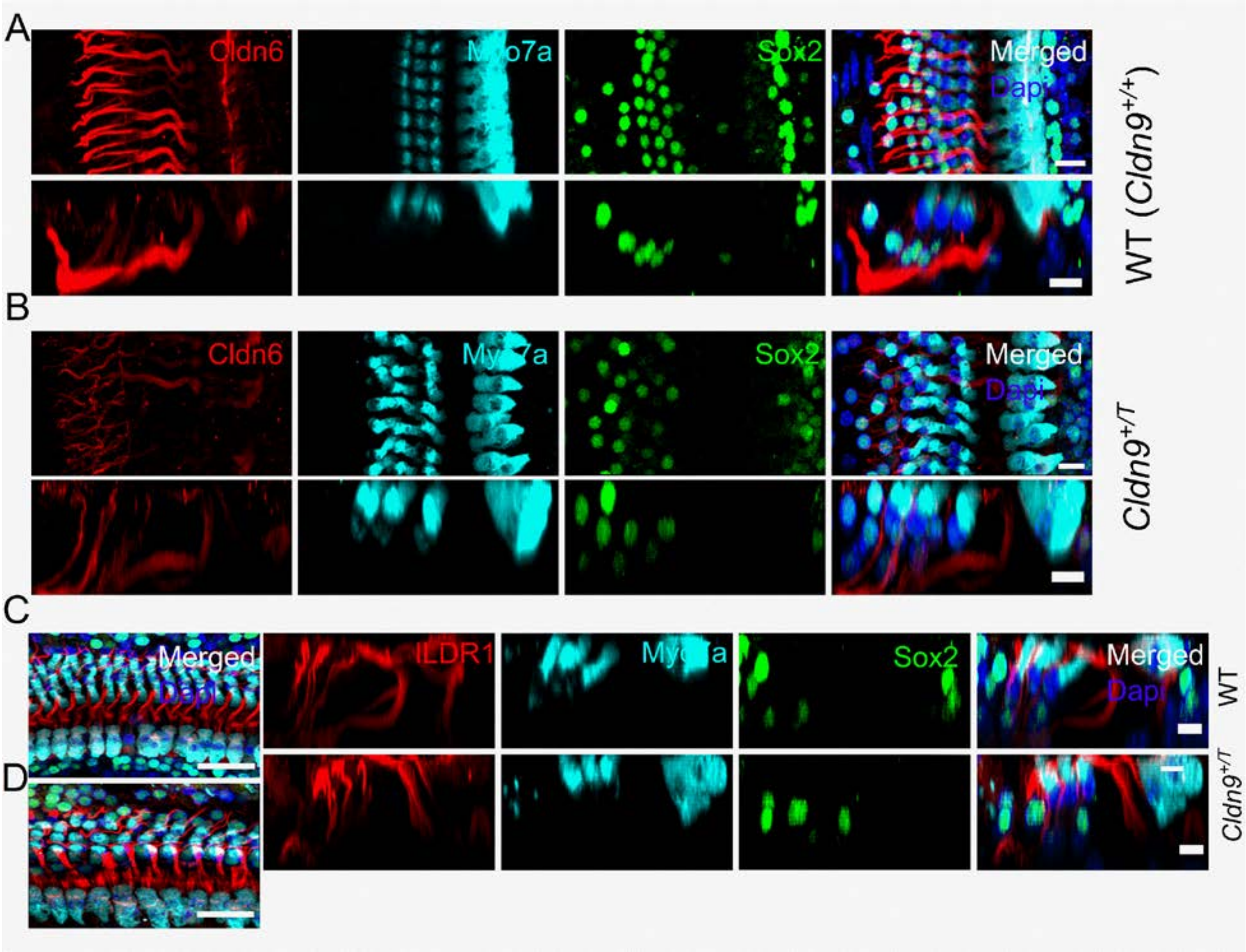

bioRxiv preprint doi: https://doi.org/10.1101/2023.10.08.561387; this version posted October 10, 2023. The copyright holder for this preprint (which was not certified by peer review) is the author/funder, who has granted bioRxiv a license to display the preprint in perpetuity. It is made available under aCC-BY 4.0 International license.

Supplement S4

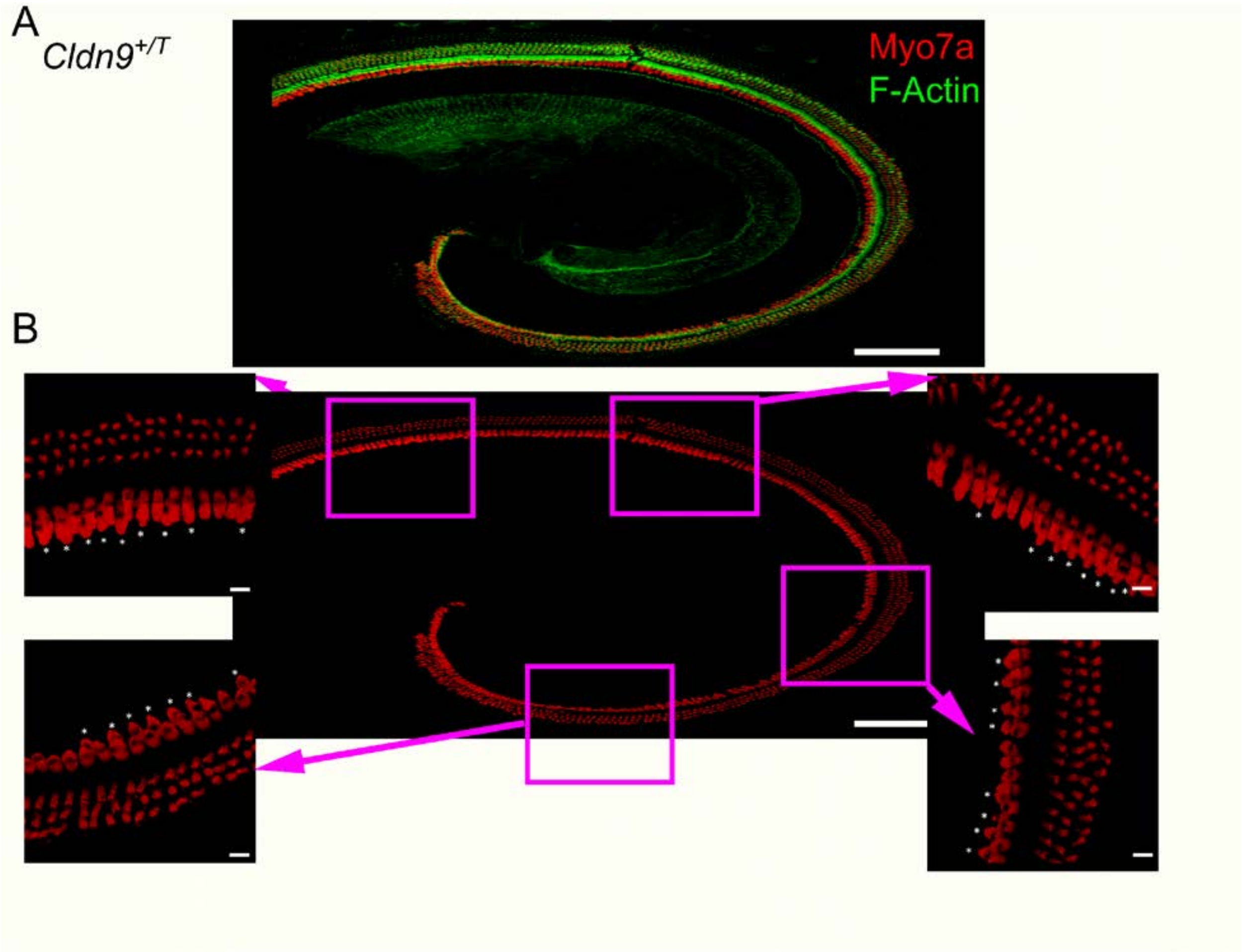

bioRxiv preprint doi: https://doi.org/10.1101/2023.10.08.561387; this version posted October 10, 2023. The copyright holder for this preprint (which was not certified by peer review) is the author/funder, who has granted bioRxiv a license to display the preprint in perpetuity. It is made available under aCC-BY 4.0 International license.

Supplement S5

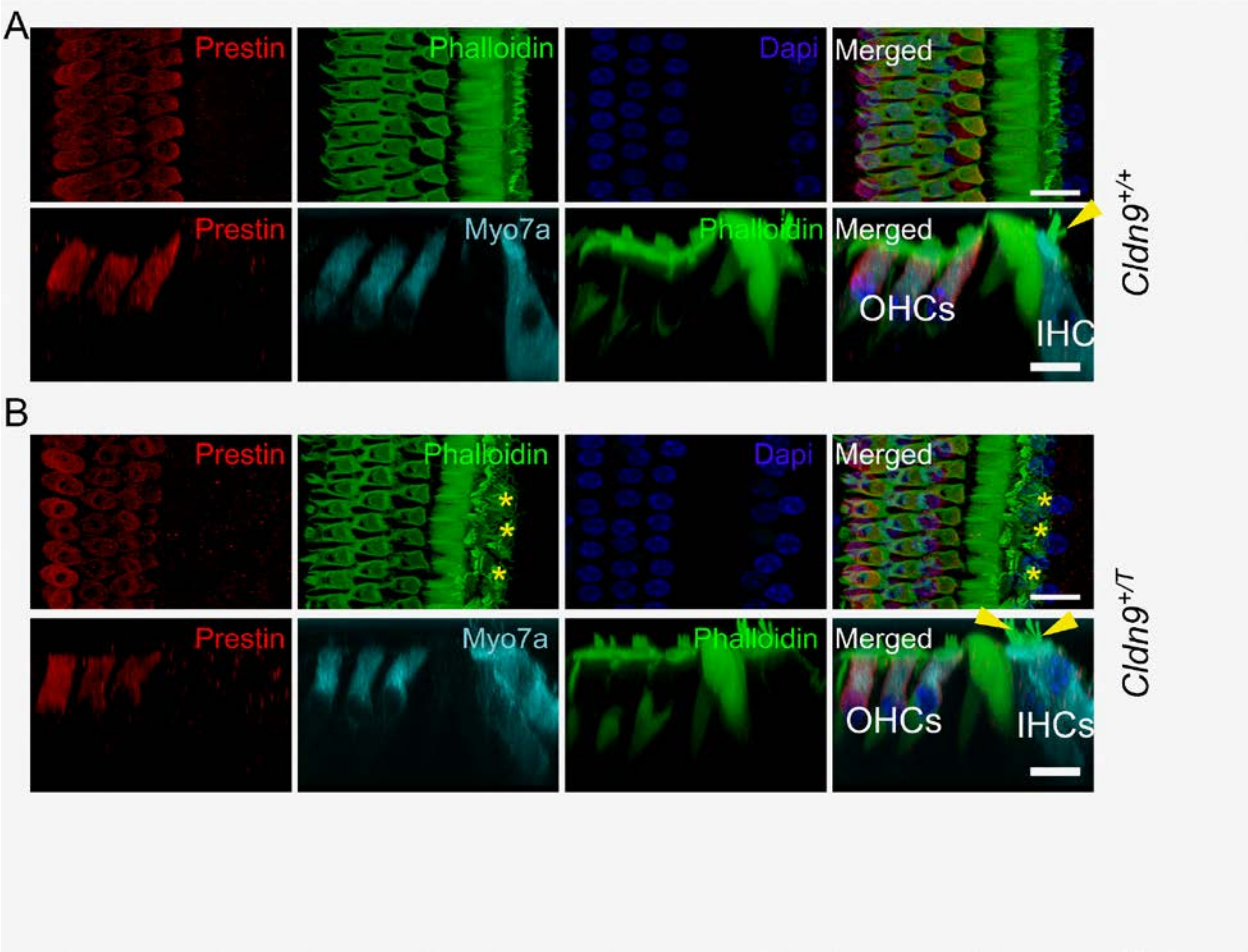

bioRxiv preprint doi: https://doi.org/10.1101/2023.10.08.561387; this version posted October 10, 2023. The copyright holder for this preprint (which was not certified by peer review) is the author/funder, who has granted bioRxiv a license to display the preprint in perpetuity. It is made available under aCC-BY 4.0 International license.

Supplement S6

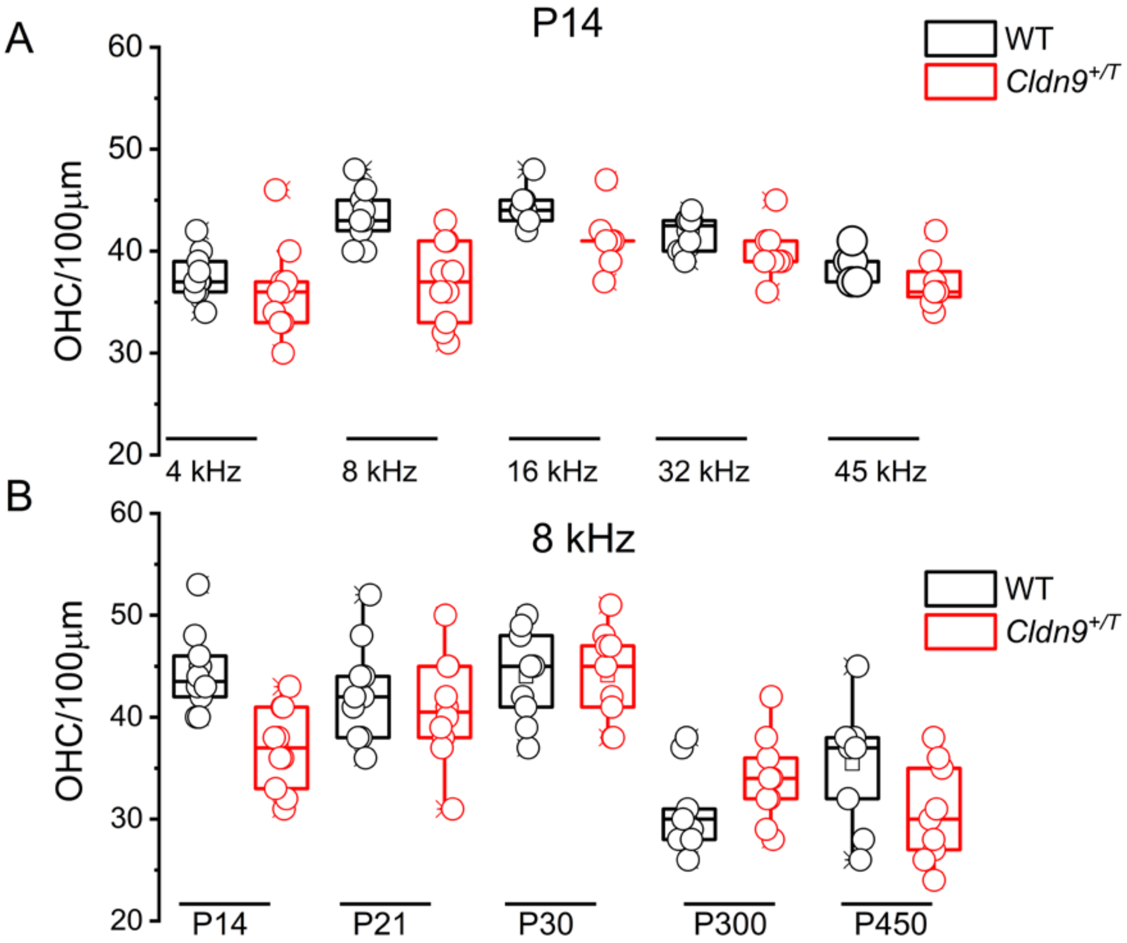

bioRxiv preprint doi: https://doi.org/10.1101/2023.10.08.561387; this version posted October 10, 2023. The copyright holder for this preprint (which was not certified by peer review) is the author/funder, who has granted bioRxiv a license to display the preprint in perpetuity. It is made available under aCC-BY 4.0 International license.

Supplement S7

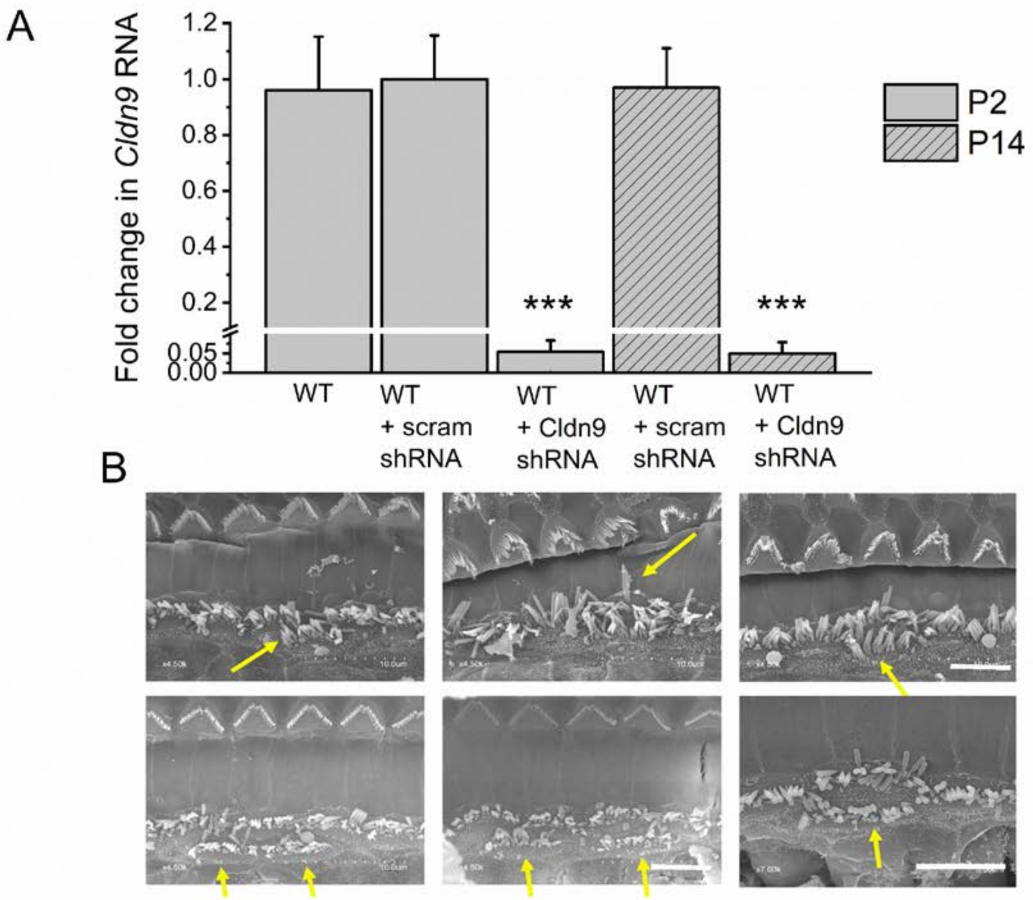

bioRxiv preprint doi: https://doi.org/10.1101/2023.10.08.561387; this version posted October 10, 2023. The copyright holder for this preprint (which was not certified by peer review) is the author/funder, who has granted bioRxiv a license to display the preprint in perpetuity. It is made available under aCC-BY 4.0 International license.

Supplement S8

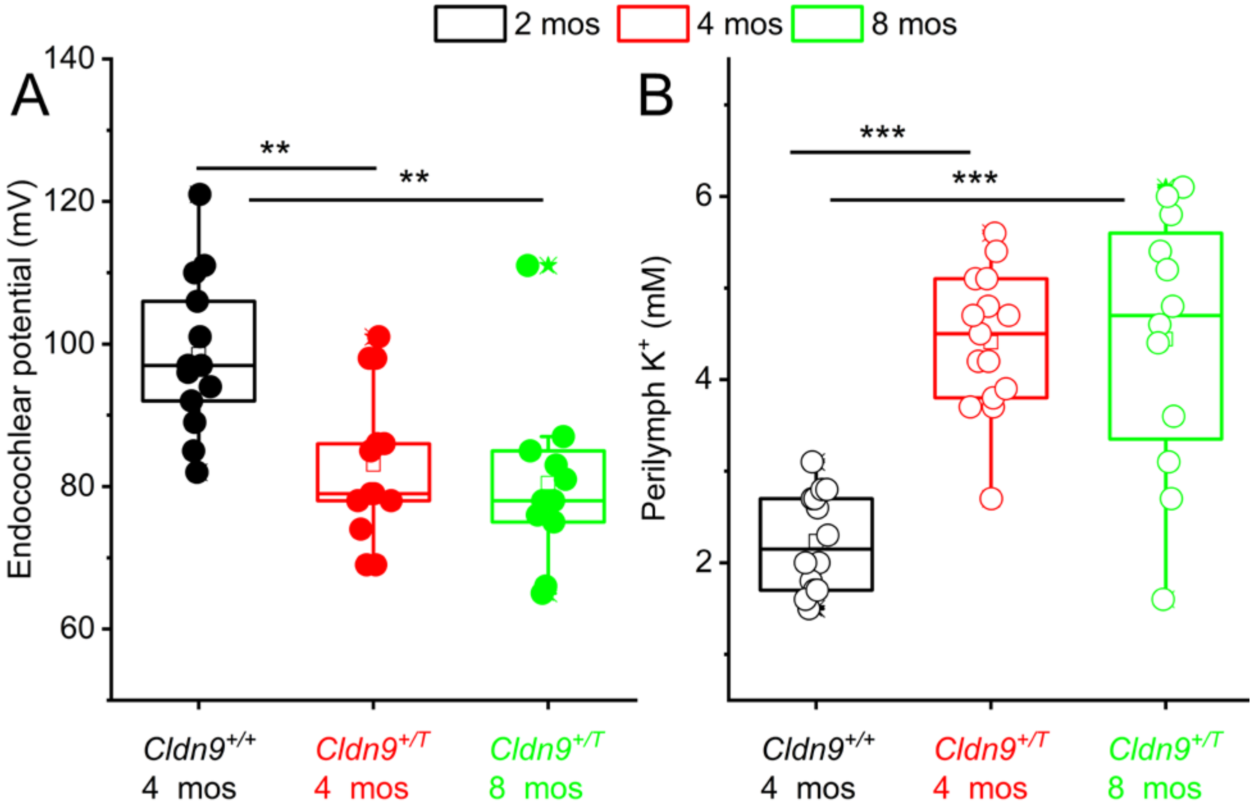

Supplement: Supplement 1 — Supplement S1 A. Quantitative RT-PCR of Cldn9 transcripts from cochlear tissue from six groups of animals, including Cldn9T/T, Cldn9+/T with and without dox treatment compared with WT littermates with and without dox treatment. B. Body weight measurements of female and male mice from dox-treated Cldn9+/T and Cldn9+/+ groups were recorded at 4, 6, and 8 wks old. C. The immunostaining of Cldn9 in 8-wk old WT (Cldn9+/+) mouse cochleaCldn9 (red), IHC stained myosin7a (cyan) and supporting cells stained Sox2 (green) and Dapi (blue) for nuclear stain. Scale = 10 μm. S2 Immunogold localization of Cldn9 in Cldn9+/T and Cldn9+/+ mice. A. Cldn9 expression sites between inner hair cells (IHCs) and supporting cells (SCs) were examined with immunogold electron microscopy with the post-embedding technique. Secondary antibodies are conjugated to 16-nm colloidal gold particles (arrows). Gold particles were noted at the junctions between SC and IHCs in Cldn9+/+ mice cochlea (indicated). Cldn9+/T mice had reduced gold particles. B. Summary of gold particle counts between IHC and SC between Cldn9+/+ and Cldn9+/T. Mean gold particles (mean±SD) for Cldn9+/+ = 25±14 (n = 41 from 3 cochleae) and for Cldn9+/T = 3±2 (n = 41, from 4 cochleae), p = 2.8X10−15. S3 The expression of cldn6 and ILDR1 in the organ of Corti. A-B, The immunostaining of Cldn6 (red) in the mouse cochlea from Cldn9+/+ (wildtype, WT) and Cldn9+/T. The lower Panel is the side view of the cochlear section. HCs were labeled with Myo7a (cyan) supporting cells with Sox2 (green) and Dapi-stained (blue) nuclei. The levels of Cldn6 were reduced in the Cldn9+/T cochlea. C-D, The immunostaining of ILDR1 (red) in the mouse cochlea from Cldn9+/+ and Cldn9+/T. The lower Panel is the side view of the cochlear section. HCs were labeled with Myo7a (cyan) supporting cells with Sox2 (green) and Dapi-stained (blue) nuclei. The levels of ILDR1 were increased in the Cldn9+/T cochlea. Scale bar = 10 μm. S4 The ectopic HCs can be seen along th [file NIHPP2023.10.08.561387v1-supplement-1.pdf]
